# The Inheritance of Mutilations and Other Inheritances

**Published:** 1897-05

**Authors:** S. M. Worthington


### THE INHERITANCE OF MUTILATIONS AND OTHER INHERITANCES.

BY S. M. WORTHINGTON, M. D.

Since the publication of Dr. Lockwood's experiments in breeding tailless mice, it rather seems accepted as true by the profession that a mutilation has but to be inflicted on an indefinite number of successive generations to insure its heredity. While this is indisputably proven regarding mice, yet facts within the observation of all cause one to doubt whether the same is true with other animals. The dehorning of cattle has been practiced for quite a time, yet I have known no hornless offspring of decornutated ancestry. My own family has occupied the same lands for nearly a century—four generations. Each in succession has marked hogs with the same mutilation of the ears. Yet none has seen a single pig born with mutilated aural appendages. And it is conceded that our stock hogs are of the same general ancestry on one side, improvement being sought by changing the sire, which also invariably suffered the same mutilation. The custom of cutting off the tails of young lambs is as old as the settlement of this State, was brought hence from Virginia, where it obtained since her colonization, coming thence from England, extending I do not know how far back, at least to the era of Little Bo-Peep. I have made careful inquiry of both breeders and buyers of sheep, and have learned of but two instances of lambs born without tails. Since I have myself observed more than a like number of tailless calves at birth, I fairly reason that these were freaks of nature rather than instances of the heredity of mutilation. These exampies afford fair grounds for belief that the mouse is less tenacious of racial characteristics than any other of the lower animals affording means of observation regarding the transmission of mutilations, if indeed they do not absolutely show that the inheritance of mutilations is a legacy peculiar to the mouse alone among all the lower animals. That there is no such inheritance in the human race, we have a proof as indisputable as can be imagined. The Jews are probably of all races the least miscegenated. They have practiced circumcision since the time of Abraham. Yet by "tale or history" we know of no offspring of that race denied the said rite by reason of congenital absence of the foreskin. While the inheritance of mutilations seems exceptional, and even disproven regarding many animals from fairly extensive observation and experiment, the transmission of mental impress is much more susceptible of proof. Mr. Railey, a noted horseman of this county, had a mare and a stallion both well-trained in the "high school" and "park" gaits. Their sires probably had received the same education; I know nothing as to their dams.

I have seen a colt of these two go "high school" in the pasture, of his own free-will and inclination, before he ever knew bit or rein. The sire in question became the property of Mrs Joe Emmet, and is, I think, to be seen in your city to-day. Another instance in this kind of heredity has been seen by all. What is known as the "pointing" or "sitting" instinct in dogs is but the development through heredity of that ecstatic pause that the presence of quarry excites in beasts of prey. It is to be observed to a slight extent in all members of both the dog and cat family. The pause or "crouch" of the lion or tiger before springing is not so much for pose of limb for that purpose as it is the transient hypnosis from imminence of prey. This hypnosis of sight or ecstatic expectancy, developed by education and fixed heredity, is surely the most reasonable explanation of the distinguishing characteristic of our bird dogs. It has had no development in the cat for sufficient reasons. She is the most uncivilized of all domestic animals. What she originally was and what she is now are one and the same, except as influenced by environment. The cat is unsusceptible of education. Such things as further her physical needs she takes advantage of, not from the influence of man, but merely as she would have done had she found the same conditions in nature. As I have seen elsewhere mentioned, she will rub her back against a man's legs as formerly against a tree, prefers the shelter of a fireside to that of a forest, food freely bestowed to the chances of the chase; but in native instincts she is in no wise changed or developed from her aboriginal state, and even in color constantly tends to the mottling of the mingling lights and shadows that proved her protection in the pristine forests.—*Medical Record.*

